# Correlation consistent auxiliary basis sets in density fitting Hartree–Fock: The atoms sodium through argon revisited

**DOI:** 10.1002/jcc.27069

**Published:** 2023-01-13

**Authors:** Harry W. Nash, Robert A. Shaw, John Grant Hill

**Affiliations:** ^1^ Department of Chemistry University of Sheffield Sheffield UK

**Keywords:** basis sets, correlation consistent, density fitting, electronic structure, high‐accuracy

## Abstract

We present a series of auxiliary basis sets, for the elements Na to Ar, for use in density‐fitted Hartree–Fock calculations with the correlation consistent cc‐pV(*n* + d)Z orbital basis sets. Benchmarking on total molecular energies, reaction energies and the spectroscopic constants of the SO molecule demonstrate that the new sets address the deficiencies of using existing auxiliary sets in combination with these orbital basis sets. We also report auxiliary basis sets for Na and Mg matched to cc‐pV*n*Z, along with recommendations for pairing auxiliary basis sets to the cc‐pV*n*Z‐F12 basis sets for Hartree–Fock calculations.

## Graphical abstract

New auxiliary basis sets for use with the correlation consistent cc‐pV(*n* + d)Z orbital basis in density fitting Hartree–Fock calculations have been developed and demonstrate significantly improved performance for total molecular and reaction energies. Recommendations for their use in the Hartree–Fock component of an explicitly correlated calculation are also made.

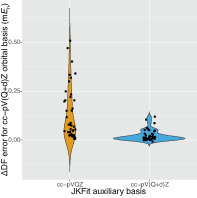

## 1 INTRODUCTION

Many electronic structure methods can use density fitting [DF, sometimes referred to as resolution‐of‐the‐identity (RI)] to approximate integrals and thereby increase computational efficiency.^1^, ^2^, ^3^ These methods include, but are not limited to, Hartree–Fock (HF),^4^ density functional theory,^5^, ^6^ complete active space self‐consistent field,^7^ and second‐order Møller–Plesset perturbation theory.^8^ In DF‐HF, the products of orbital basis functions are approximated using auxiliary basis functions in the evaluation of both the Coulomb (**J**) and exchange (**K**) matrices. It has been demonstrated that auxiliary basis sets fit against the exchange matrix can also be used in the fitting of the Coulomb integrals,^4^ with the resulting auxiliary basis sets suffixed JKFit. Such auxiliary sets have been optimized for use with various orbital basis sets and are available for many elements of the Periodic Table.^4^, ^9^ Automated methods for the generation of auxiliary basis sets have also been proposed,^10^, ^11^, ^12^ with some of these implemented such that they produce an auxiliary basis set on‐the‐fly as part of an electronic structure calculation. While such automated methods are both convenient and accurate, the auxiliary sets generated in this way tend to be larger than those optimized using the traditional, “manual” approach, increasing computational cost.

DF‐HF is often used to provide the reference wavefunction for local correlation methods, which aim to reduce the steep scaling of computational effort with respect to system size by exploiting the local nature of electron correlation. A detailed review of such methods is beyond the scope of this article, but a number of different approaches to using locality for reduced scaling are the subject of a recent book.^13^ Of particular interest here are explicitly correlated (F12) local coupled cluster methods using pair natural orbitals,^14^ such as the PNO‐LCCSD(T)‐F12 or $\text{DLPNO}‐\text{CCSD}{\left(T\right)}_{\bar{F12}}$ methods.^15^, ^16^ By including terms that depend explicitly on inter‐electronic distance, modern F12 methods significantly reduce the basis set incompleteness error (BSIE) in the correlation energy,^17^, ^18^, ^19^, ^20^ and reduce the domain error associated with local methods.^21^, ^22^ This means that the density fitting error at the Hartree–Fock level, which is usually insignificant with canonical correlation treatments, can become an important source of error in the explicitly correlated local correlation case.^23^

The correlation consistent polarized valence *n*‐zeta (cc‐pV*n*Z) basis sets are often used in high‐accuracy wavefunction‐based calculations,^24^ partially due to their construction that systematically approaches the complete basis set (CBS) limit as *n* is increased.^25^ For the second‐row elements Al‐Ar,^26^ it was first noted by Bauschlicher and Partridge,^27^ and subsequently expanded upon by Martin,^28^, ^29^ that unacceptably high errors in binding energies could result from using the cc‐pV*n*Z basis sets, even at the HF level. The inclusion of an additional “tight” (large exponent) d‐exponent was later demonstrated to ameliorate this problem,^30^ and the resulting cc‐pV(*n* + d)Z basis sets should be used for calculations involving the elements aluminum through argon. More recent correlation consistent basis sets for these elements, such as the cc‐pV*n*Z‐F12 family designed for explicitly correlated calculations,^31^ include the tight‐d functions as a matter of course.

The cc‐pV*n*Z‐JKFit auxiliary basis sets of Weigend^4^ (where the ‐JKFit suffix indicates these are auxiliary basis sets for use in DF‐HF calculations) are commonly used in DF‐HF calculations with either the cc‐pV(*n* + d)Z or cc‐pV*n*Z‐F12 orbital sets, but these auxiliary bases were not optimized for use with correlation consistent basis sets that include the additional tight‐d functions. Werner and co‐workers noted that this can lead to large errors, particularly when sulfur atoms are present. For example, using the aug‐cc‐pVTZ‐JKFit auxiliary set in combination with F12 specific basis sets leads to a fitting error in the Hartree–Fock energy of 0.28 kcal mol^−1^ for the reaction $C_{4}H_{9}{\mathrm{SO}}_{2}H+H_{2}O_{2}\to C_{4}H_{9}{\mathrm{SO}}_{3}H+H_{2}O$,^23^ which is reaction 4 of the test set of 51 reaction energies proposed by Friedrich and Hänchen (referred to as the FH test set herein).^32^ The addition of an ad hoc tight f‐function and one g‐function to the JKFit basis for sulfur reduced this error to 0.03 kcal mol^−1^,^23^ and indicates that revising the JKFit basis sets to account for the tight‐d function is important for high‐accuracy calculations. We also note that the def2‐QZVPP‐JKFit sets of Weigend were fit to orbital basis sets that do contain tight‐d functions,^9^ and are used as the default auxiliary basis for DF‐HF in some electronic structure codes, even when a cc‐pV(*n* + d)Z orbital basis is selected.

In this contribution, we detail the optimization and benchmarking of new JKFit basis sets for sodium and magnesium, and revisit the JKFit sets for aluminum through argon to ensure robust density fitting when correlation consistent basis sets that include tight‐d functions are used.

## 2 METHODOLOGY

### 2.1 General computational procedure

All electronic structure calculations in this work were carried out using the Molpro system of ab initio programs.^33^, ^34^ A modified version of the code was used to obtain the density fitted density matrix and exchange integrals required to optimize the exponents of the auxiliary basis sets, with BFGS (using numerical gradients) or Nelder–Mead simplex algorithms employed in the optimization.^35^, ^36^ For the atoms aluminum through argon the cc‐pV(*n* + d)Z orbital basis sets were used,^30^ and the cc‐pV*n*Z‐JKFit auxiliary basis sets of Weigend were the starting point for the auxiliary basis set optimization.^4^ For sodium and magnesium, new cc‐pV*n*Z‐JKFit auxiliary sets matched to the corresponding orbital sets were optimized in this work,^37^ along with the analogous cc‐pV(*n* + d)Z‐JKFit auxiliary sets.

For the benchmarking of the auxiliary sets we define the density fitting error, ΔDF, as the difference between the total energy calculated with DF‐HF and that with conventional HF. When molecules contain first‐row elements (or hydrogen) the cc‐pV*n*Z orbital basis sets were used for those elements,^24^ along with the matching cc‐pV*n*Z‐JKFit auxiliary sets.^4^ To place ΔDF in context, the BSIE of the molecules for the orbital basis sets has been calculated from CBS limits estimated with the extrapolation formula of Karton and Martin,^38^ using the HF cc‐pVQZ and cc‐pV5Z energies. We note that the intention here is not to establish highly‐accurate CBS limits, but the order of magnitude difference between BSIE and ΔDF. The density fitting error was also assessed for the scenario where the cc‐pV(*n* + d)Z‐JKFit auxiliary sets are used in conjunction with the cc‐pV*n*Z‐F12 orbital basis sets.^31^, ^39^ The density fitting error in the dissociation energy (*D*_e_), equilibrium bond length (*r*_e_), and harmonic frequency (*ω*_e_) of the diatomic molecule SO at the HF level of theory was obtained by calculating the energy of seven near‐equilibrium points (−0.3*a*_0_ ≤ *r* − *r*_e_ ≤ +0.5*a*_0_) and fitting them with a sixth‐order polynomial before a Dunham analysis.^40^

### 2.2 Auxiliary basis set optimization

The optimization of exponents within the JKFit auxiliary sets developed in this work closely followed the previous work of Weigend.^4^, ^9^ All functions are uncontracted and the exponents were optimized to minimize the error in the HF exchange energy:

(1) $$\delta_{\mathrm{EX}}=\left|\mathrm{Tr}\left\{P^{\mathrm{DF}}K^{\mathrm{DF}}\right\}-\mathrm{Tr}\left\{P^{\text{Full}}K^{\text{Full}}\right\}\right|,$$

where P and K are the density matrix and exchange integrals, respectively. The superscripts DF and Full indicate whether they were calculated using density fitting or full integrals. For further details of the DF‐HF method and suggested goals for the development of JKFit auxiliary sets, the interested reader is referred to the work of Weigend and references therein.^4^, ^9^

To develop cc‐pV*n*Z‐JKFit auxiliary sets for Na and Mg, optimizations were carried out on NaH and MgH^+^, respectively. Briefly, a common (13s11p9d) auxiliary basis “kernel” was optimized for the cc‐pV5Z orbital basis and used in all JKFit sets for these elements. Higher‐angular momentum functions for TZ–5Z were optimized for the corresponding orbital basis, with the total composition of the resulting JKFit sets (see Table 1) matching those of Weigend for the elements Al–Cl.^4^ Following the procedure adopted in the Molpro basis set library,^33^ cc‐pVDZ‐JKFit sets for Na and Mg were generated by simply removing the g‐type functions from cc‐pVTZ‐JKFit.

**TABLE 1:** Composition of the JKFit basis sets developed in this work [cc‐pV(*n* + d)Z‐JKFit] compared to the existing cc‐pV*n*Z‐JKFit. The new auxiliary basis sets add new 1f1g exponents (1f only at the DZ level) and re‐optimize all existing f exponents

Orbital basis	cc‐pV*n*Z‐JKFit	cc‐pV(*n* + d)Z‐JKFit
cc‐pV(D + d)Z	(13s11p9d3f)	(13s11p9d4f)
cc‐pV(T + d)Z	(13s11p9d3f1g)	(13s11p9d4f2g)
cc‐pV(Q + d)Z	(13s11p9d4f2g1h)	(13s11p9d5f3g1h)
cc‐pV(5 + d)Z	(13s11p9d4f3g2h1i)	(13s11p9d5f4g2h1i)

As the cc‐pV(*n* + d)Z orbital basis sets are obtained from cc‐pV*n*Z with an additional d‐function and a re‐optimization of the existing d‐function exponents, we approach the development of the cc‐pV(*n* + d)Z‐JKFit auxiliary sets by modifying the cc‐pV*n*Z‐JKFit sets. This was also motivated by the promising results of Ma et al. for sulfur.^23^ Initial testing revealed that the addition of s‐, p‐ or d‐type auxiliary functions to cc‐pV*n*Z‐JKFit had effectively zero effect on both *δ*_EX_ and the total DF‐HF energy. The same was also true for higher orbital angular momentum auxiliary functions beyond g‐type, hence we restricted any modifications to the auxiliary basis sets to the f‐ and g‐type functions.

Attempting to optimize new f‐type exponents while keeping the existing exponents fixed resulted in functions that were too diffuse to be effective in reducing the density fitting error; in most cases the additional exponents led to an increase in ΔDF. Adding an additional f‐ and g‐type exponent, and then re‐optimizing all of the f‐exponents for (diatomic) monohydrides instead proved to reduce both *δ*_EX_ and ΔDF to negligible values, with the largest f‐type exponents being significantly tighter than those when only the tightest exponents were optimized.

Again matching the Molpro basis set library, the new exponents for Ar were determined as a “constant‐shift” extrapolation based on the exponents for S and Cl. Concretely, *ζ*_Ar_ = (*ζ*_Cl_ − *ζ*_S_) + *ζ*_Cl_. The final compositions of the cc‐pV(*n* + d)Z‐JKFit auxiliary basis sets are presented in Table 1, where a comparison with cc‐pV*n*Z‐JKFit confirms the small number of additional exponents added to “fit” the tight‐d functions in the orbital basis set. The analogous def2‐QZVPP‐JKFit auxiliary sets of Weigend are composed of (16s12p10d4f1g) primitives contracted to [13s11p9d4f1g].^9^ The contracted form of this auxiliary basis is almost the same composition as the uncontracted cc‐pV(T + d)Z‐JKFit presented in this work, and does not contain the higher orbital angular momentum functions found in cc‐pV(Q + d)Z‐JKFit or cc‐pV(5 + d)Z‐JKFit.

JKFit sets for use with diffuse augmented correlation consistent orbital basis sets, the latter often denoted aug‐cc‐pV*n*Z and aug‐cc‐pV(*n* + d)Z,^30^, ^37^, ^41^ were created by augmenting the cc‐pV(*n* + d)Z‐JKFit sets detailed above with an additional exponent for each orbital angular momentum shell. The exponents were determined using the even‐tempered procedure $\zeta_{\mathrm{aug}}=\zeta_{1}^{2}/\zeta_{2}$, where *ζ*_1_ is the most diffuse exponent (of the same orbital angular momentum) present in the parent JKFit basis, and *ζ*_2_ is the next most diffuse exponent. In cases where there is only a single exponent of a given orbital angular momentum present, the augmenting exponent was determined as *ζ*_aug_ = *ζ*_1_/2.5. These even‐tempered procedures match those used to generate the aug‐cc‐pV*n*Z‐JKFit sets in the Molpro basis set library.^33^ Due to the nature of the auxiliary basis set modifications required to optimize the cc‐pV(*n* + d)Z‐JKFit sets detailed above, for Al–Ar this means that only the f‐type diffuse exponent changes relative to the existing aug‐cc‐pV*n*Z‐JKFit. The compositions of the diffuse augmented auxiliary sets are presented in Table S1 in Data S1.

## 3 RESULTS AND DISCUSSION

### 3.1 Molecular energies

#### 3.1.1 cc‐pV*n*Z‐JKFit sets for Na and Mg

For the elements sodium and magnesium, where there are no previous JKFit sets specifically matched to the correlation consistent orbital bases, the error due to using the cc‐pV*n*Z‐JKFit sets developed in this work has been determined for the molecules Mg_4_, MgCl_2_, MgF, MgH_2_, Na_2_O, Na_2_S, Na_3_N, Na_3_P, NaCl, NaF and NaH. The geometries of these molecules were obtained from the work of Weigend.^9^ The mean absolute error (MAE), standard deviation (*σ*) and maximum absolute error (MAX) in terms of ΔDF are summarized for each *ζ*‐level in Table 2. Also shown is the conventional HF BSIE as summary statistics for the same molecules. It can be seen that as the *ζ*‐level of the basis set increases, the error due to the density fitting decreases. The values of *σ* and MAX for ΔDF are relatively similar for DZ and TZ, which may be due to the similarity of the respective JKFit sets (they are identical, bar TZ containing g functions), but the density fitting errors are negligible at two to three orders of magnitude smaller than the equivalent BSIE.

**TABLE 2:** Orbital basis set incompleteness error (BSIE) and error due to density fitting (ΔDF) in the Hartree–Fock energies for a test set of molecules containing Na and Mg. All errors are presented in m*E*_h_

Error type	Basis set	MAE	*σ*	MAX
Orbital BSIE	cc‐pVDZ	40.70616	25.83619	106.87881
	cc‐pVTZ	11.02122	7.21668	28.89166
	cc‐pVQZ	3.39788	1.92311	7.09940
	cc‐pV5Z	0.70625	0.39834	1.49704
ΔDF	cc‐pVDZ‐JKFit	0.05579	0.03468	0.10450
	cc‐pVTZ‐JKFit	0.03992	0.02588	0.09243
	cc‐pVQZ‐JKFit	0.01089	0.00866	0.02970
	cc‐pV5Z‐JKFit	0.00879	0.00678	0.02326

#### 3.1.2 Tight‐d functions in JKFit sets

To determine the efficacy of the new auxiliary basis sets in reducing ΔDF in molecular energies, first we estimate conventional HF/CBS limits for a test set of molecules containing second‐row elements, and use these to establish BSIEs for both the cc‐pV(*n* + d)Z and cc‐pV*n*Z‐F12 orbital basis set families. The test set contains 41 molecules selected from the larger test set of Weigend that was previously used in assessing density fitting errors at the HF level,^9^ with further details of the test set provided in Data S1. The resulting BSIEs are presented in Table 3 as MAE, *σ* and MAX for each basis set. As expected from correlation consistent basis sets, there is systematic convergence toward the estimated HF/CBS limit, with the error reducing by roughly a factor of five with each *ζ*‐level. The cc‐pV*n*Z‐F12 basis sets have BSIEs approximately equal to that of cc‐pV([*n* + 1] + d)Z, but this is to be expected as the F12 specific basis sets use the s and p primitives from the cc‐pV(*n* + 1)Z conventional sets and contain tight‐d functions for second‐row elements. The motivation for this design choice was to avoid basis set errors in F12 total energies being dominated by the HF component,^31^ and it also suggests that F12 specific basis sets may require a different JKFit auxiliary basis than for a conventional correlation consistent basis set of the same *ζ*‐level.

**TABLE 3:** Orbital basis set incompleteness error in Hartree–Fock energies for a test set of 41 second‐row element containing molecules with both the cc‐pV(*n* + d)Z and cc‐pV*n*Z‐F12 basis set families. All errors are presented in m*E*_h_

Basis set family	*ζ*‐level	MAE	*σ*	MAX
cc‐pV(*n* + d)Z	DZ	88.54652	84.15989	339.01456
	TZ	20.05070	18.90737	76.18122
	QZ	4.29129	4.22692	17.56386
	5Z	0.74031	0.67245	2.51822
cc‐pV*n*Z‐F12	DZ	25.62360	24.72747	106.61486
	TZ	4.35712	4.32721	18.28461
	QZ	0.51266	0.52339	2.07156

**FIGURE 1:**
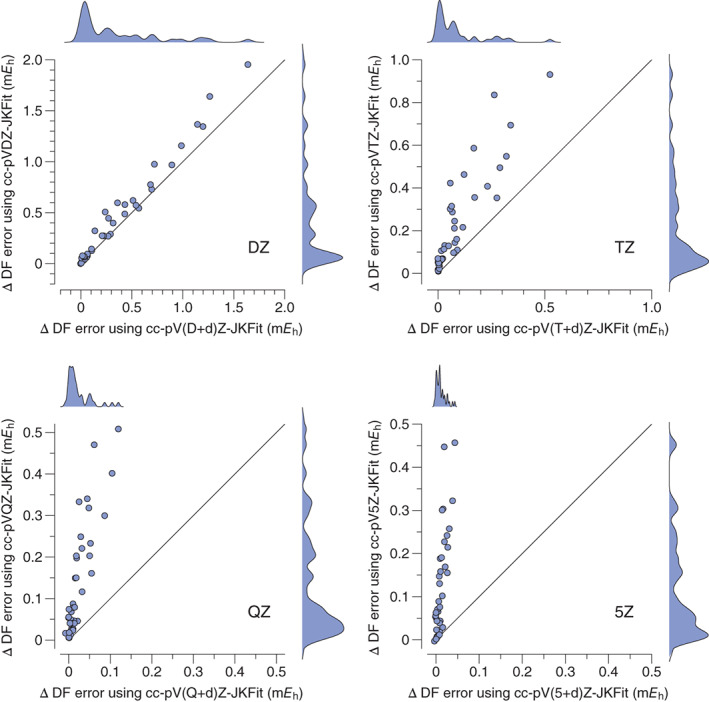
ΔDF errors (m*E*_h_) per molecule for the cc‐pV(*n* + d)Z basis sets using the cc‐pV*n*Z‐JKFit and cc‐pV(*n* + d)Z‐JKFit auxiliary sets. Horizontal distance from the solid diagonal line indicates the relative improvement when using cc‐pV(*n* + d)Z‐JKFit. Each plot in the grid also shows the distribution of the ΔDF errors for each JKFit auxiliary basis as smoothed histograms

The MAE due to density fitting with the auxiliary basis sets developed in this work is compared to that from using the cc‐pV*n*Z‐JKFit and def2‐QZVPP‐JKFit auxiliary sets in Table 4 for the same test set of 41 molecules. The values of *σ* and MAX are tabulated in Data S1, where violin plots showing the distribution of ΔDF can also be found. For all *ζ*‐levels there is a reduction in error when using the new cc‐pV(*n* + d)Z‐JKFit sets and the improvement in the accuracy of the density fitting increases with basis set cardinal number. At the DZ level there is a relatively modest improvement, which increases to a whole order of magnitude improvement at the 5Z level. The comparison between the cc‐pV*n*Z‐JKFit and cc‐pV(*n* + d)Z‐JKFit sets are also presented as scatter plots and smoothed histograms in Figure 1. The scatter plots include a solid diagonal line that indicates where the two auxiliary sets would produce the same density fitting error. Bar a very small number of DZ cases, the points all lie on the left of the diagonal line, indicating that the cc‐pV(*n* + d)Z‐JKFit auxiliary set has a smaller density fitting error for that molecule. This is particularly striking in the QZ and 5Z cases, where the new basis sets have a very tight distribution of the errors.

**TABLE 4:** Comparison of the mean absolute errors (m*E*_h_) due to different auxiliary basis sets in the density fitting of HF energies for a test set of 41 second‐row element containing molecules. The cc‐pV(*n* + d)Z orbital basis set is used in all cases

*ζ*‐level	cc‐pV*n*Z‐JKFit	cc‐pV(*n* + d)Z‐JKFit	def2‐QZVPP‐JKFit
DZ	0.44386	0.36047	0.11550
TZ	0.22923	0.09221	0.11449
QZ	0.13433	0.02372	0.14117
5Z	0.11731	0.01112	0.15202

The performance of the existing cc‐pV*n*Z‐JKFit and new cc‐pV(*n* + d)Z‐JKFit sets can also be compared to that of def2‐QZVPP‐JKFit, which is used as a form of “universal” fitting set in some electronic structure codes. Focusing on the cc‐pV(D + d)Z orbital basis, it can be seen from Table 4 that using def2‐QZVPP‐JKFit results in a significantly lower MAE, and Table S2 and Figure S1 of Data S1 show that this improved fitting extends to the distribution of errors. However, it should also be noted that the def2‐QZVPP‐JKFit auxiliary set contains g‐type functions that are not present in either cc‐pVDZ‐JKFit or cc‐pV(D + d)Z‐JKFit. To determine the impact of this single set of g functions, Figure S1 also shows the distribution of ΔDF when the g functions are removed; it can be seen that the distribution becomes similar to that of cc‐pV(D + d)Z‐JKFit at that point, indicating that the improved performance is mostly due to the presence of the g‐type functions.

As the orbital basis set quality rises to TZ or beyond, Table 4 shows that the new cc‐pV(*n* + d)Z‐JKFit results in a lower MAE than def2‐QZVPP‐JKFit, with the latter producing slightly larger mean errors than even the existing cc‐pVQZ‐JKFit or cc‐pV5Z‐JKFit. Table S3 of Data S1, along with Figure S2, suggest that at the TZ orbital basis level the distribution of DF errors with cc‐pV(T + d)Z‐JKFit and def2‐QZVPP‐JKFit are quite similar and there is little to choose between them. The two auxiliary basis sets have a similar basis set composition and the exponents span a similar range, hence this is not a surprising result. With the QZ orbital basis, the new cc‐pV(Q + d)Z‐JKFit basis produces error statistics that are a factor of five smaller than those from def2‐QZVPP‐JKFit (see Table S4 and Figure S3), while at the 5Z orbital basis level, the cc‐pV(Q + d)Z‐JKFit errors are at least an order of magnitude smaller than def2‐QZVPP‐JKFit (Table S5 and Figure S4). This is presumably due to the lack of higher orbital angular momentum functions in the def2‐QZVPP‐JKFit auxiliary basis; as the def2‐QZVPP orbital basis has g‐type functions as the highest angular momentum present,^42^ def2‐QZVPP‐JKFit was not originally designed to fit orbital sets with high orbital angular momentum functions, such as cc‐pV(5 + d)Z. This leads to a slight increase in ΔDF errors from using def2‐QZVPP‐JKFit as the zeta‐level of the orbital basis set is increased, which is visualized as violin plots in Figure S5 in Data S1.

The MAE of ΔDF in Table 4 can be contrasted with the orbital BSIE in Table 3 to place values in context. For the existing cc‐pV*n*Z‐JKFit auxiliary sets it is apparent that the density fitting error is around two orders of magnitude smaller than the orbital BSIE at the DZ and TZ level, but this falls to one order of magnitude or less for QZ and 5Z. The 5Z results in particular are concerning as the error from density fitting begins to approach the error in the orbital basis set. The same is true if the def2‐QZVPP‐JKFit auxiliary basis is used along with the 5Z orbital basis. The picture is much improved when switching to the cc‐pV(*n* + d)Z‐JKFit sets of this work as the MAE becomes roughly two orders of magnitude smaller than the orbital BSIE in all cases, indicating negligible errors in the density fitting for all molecules considered.

Table 4 suggests that the cc‐pV(D + d)Z‐JKFit auxiliary basis, which adds only a single set of f‐type functions, provides only a modest improvement over cc‐pVDZ‐JKFit. This raises the question of how the accuracy of the density fitting could be improved, and whether such improvements offset any increased computational effort they would require. As the TZ‐5Z auxiliary sets add both additional f‐ and g‐type functions, we investigate the effect of adding a single set of g‐type functions to the cc‐pV(D + d)Z‐JKFit auxiliary basis [denoted cc‐pV(D + d)Z + 1 g‐JKFit herein]. This g‐function takes the exponent of the new g‐function added to cc‐pV(T + d)Z‐JKFit auxiliary basis without any re‐optimization, and results in a MAE of 0.25528 m*E*_h_ over the test set of molecules, compared to the value of 0.36047 m*E*_h_ for cc‐pV(D + d)Z‐JKFit. The standard deviation and MAX (see Data S1) are also reduced, but the improvements are small compared to the orbital BSIE (∼88.5 m*E*_h_) and the increase in computational effort of including a higher orbital angular momentum shell does not appear warranted for HF calculations of molecular energies.

To continue the discussion of efficiency of DF‐HF and how this is affected by the choice of JKFit auxiliary basis, Table 5 presents the CPU times taken for a single‐point DF‐HF energy on cyclo‐octasulfur (S_8_). Each CPU time is the mean average of three identical calculations, all run on a single core of an Intel Xeon E5‐2640. Symmetry was set to *C*_1_ for all calculations and the conventional HF timings are also provided. The results for the cc‐pV(D + d)Z orbital basis indicate that DF‐HF provides little advantage over conventional HF with this size of basis set, but the efficiency of DF‐HF does increase significantly with basis set size. For a given orbital basis set, the DF‐HF CPU time scales approximately linearly with the total number of functions in the auxiliary basis, that is, the average CPU time divided by the number of functions produces roughly the same value for all JKFit sets tested. As the cc‐pV(*n* + d)Z‐JKFit sets contain only one additional set of f‐ and g‐type functions compared to cc‐pV*n*Z‐JKFit (only one set of f‐type functions in the DZ case) it is not surprising that the new auxiliary sets require only a minor increase in CPU time. This increase is easily justified given the increases in fitting accuracy detailed above. The timings with the def2‐QZVPP‐JKFit auxiliary basis sets are also provided in Table 5 for comparison. Again, given the composition of the auxiliary basis sets, it is unsurprising that def2‐QZVPP‐JKFit requires slightly lower CPU times at the QZ and 5Z levels.

**TABLE 5:** CPU times (s) for a single‐point DF‐HF energy evaluation on S_8_ using various JKFit auxiliary basis sets. The conventional HF CPU time is provided for comparison

Orbital basis	HF	DF‐HF		
		cc‐pV*n*Z‐JKFit	cc‐pV(*n* + d)Z‐JKFit	def2‐QZVPP‐JKFit
cc‐pV(D + d)Z	25.5	16.3	17.3	19.8
cc‐pV(T + d)Z	209.8	36.0	41.1	39.5
cc‐pV(Q + d)Z	1678.2	104.4	116.2	89.1
cc‐pV(5 + d)Z	11302.3	258.9	283.1	177.8

As the errors due to density fitting using the def2‐QZVPP‐JKFit auxiliary basis are of the same order as the BSIE when using the cc‐pV(5 + d)Z orbital basis set, herein we shall consider only the differences in energies and spectroscopic constants with the existing cc‐pV*n*Z‐JKFit and new cc‐pV(*n* + d)Z‐JKFit auxiliary sets used in the density fitting.

#### 3.1.3 Choice of JKFit basis in F12 calculations

The choice of JKFit auxiliary basis to pair with the cc‐pV*n*Z‐F12 orbital basis sets in F12 calculations has some additional considerations; there are currently no JKFit sets specifically matched to this family of orbital basis sets and, as seen in Table 3, the construction of the orbital sets means they have significantly reduced BSIE compared to cc‐pV*n*Z. One option would be to develop new JKFit sets specifically for use with F12 basis sets, but given that the s and p primitives in the orbital basis are taken from the cc‐pV(*n* + 1)Z conventional sets it is feasible that an acceptable level of density fitting error may be achievable using an existing auxiliary basis set. Here we consider only the effect of the fitting basis on the DF‐HF energy, any effects of the complementary auxiliary basis set (CABS) singles correction on the energy,^43^ or the possible effect of the JKFit basis on the integrals arising in F12 theory are neglected.

For each of the cc‐pV*n*Z‐F12 (*n* = D, T, Q) orbital sets, the MAE, *σ* and MAX have been calculated for the test set of 41 molecules when using the following auxiliary sets: cc‐pV*n*Z‐JKFit, cc‐pV(*n* + d)Z‐JKFit, cc‐pV[*n* + 1]Z‐JKFit and cc‐pV([*n* + 1] + d)Z‐JKFit. The same summary error statistics have also been computed for the diffuse augmented versions of those auxiliary basis sets, and for the DZ orbital basis the cc‐pV(D + d)Z + 1 g‐JKFit set. These statistics, along with the number of auxiliary basis functions for a single second‐row atom (*N*_ABS_), are presented in Tables S5–S7 in Data S1. For our recommendations, we establish a guiding principle that the summary statistics of ΔDF should be approximately two orders of magnitude smaller than the BSIE of the orbital sets presented in Table 3, while keeping *N*_ABS_ small for reasons of computational efficiency.

For the cc‐pVDZ‐F12 orbital set, sufficient accuracy in the density fitting can be attained with cc‐pV(D + d)Z + 1g‐JKFit. However, this auxiliary basis is larger than cc‐pVTZ‐JKFit and produces slightly larger errors, thus it does not represent a logical choice. In the majority of cases, augmenting the auxiliary sets with diffuse functions leads to only minor improvements and is difficult to recommend, although we do note that none of the molecules in the test set is likely to have diffuse electron distributions. For the cc‐pVTZ‐F12 orbital set, the smallest auxiliary basis to reach the two orders of magnitude threshold is cc‐pV(Q + d)Z‐JKFit and, as the orbital basis uses the s and p primitives from cc‐pVQZ, there is some underlying additional justification for this choice. To simplify the selection of DF‐HF auxiliary set for the cc‐pV*n*Z‐F12 family, the cc‐pV([*n* + 1] + d)Z‐JKFit sets represent a reasonable balance between cost and accuracy. For cc‐pVQZ‐F12 this produces errors that are slightly less than two orders of magnitude smaller than the orbital BSIE, but the only auxiliary basis that produces more accurate results is aug‐cc‐p(5 + d)Z‐JKFit, which possesses around 25% more auxiliary basis functions.

**FIGURE 2:**
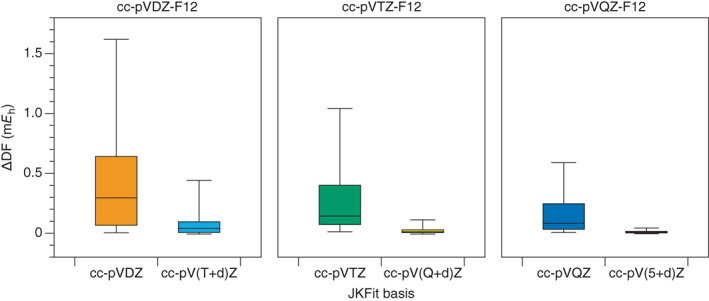
Comparison of cc‐pV*n*Z‐JKFit and cc‐pV([*n* + 1] + d)Z‐JKFit auxiliary sets in terms of the HF density fitting error (m*E*_h_) when used in conjunction with the cc‐pV*n*Z‐F12 orbital sets. The errors are in molecular energies for a test set of 41 second‐row element containing molecules. The whiskers on each box plot indicate the minimum and maximum errors

Figure 2 compares the DF error in the total HF energy for the recommended cc‐pV([*n* + 1] + d)Z‐JKFit set with the cc‐pV*n*Z‐JKFit set that may serve as a default choice. It is clear that choosing cc‐pV([*n* + 1] + d)Z‐JKFit leads to a significant reduction in error at all *ζ*‐levels, and that the error reduces with orbital basis set cardinal number. For TZ and QZ orbital sets, the boxes corresponding to the cc‐pV([*n* + 1] + d)Z‐JKFit errors become squashed, reflecting both the very small average errors and the small range of error.

### 3.2 Reaction energies

While molecular energies are convenient for the purposes of benchmarking the error due to density fitting, there is a possibility that any fitting errors cancel once relative energies are considered. Here we consider the seven reactions from the FH test set that contain second‐row elements and determine the error in the HF reaction energies due to the density fitting approximation. The selected reactions are:

$$C_{4}H_{9}{\mathrm{SO}}_{2}H+H_{2}O_{2}\to C_{4}H_{9}{\mathrm{SO}}_{3}H+H_{2}O,$$

$$S{\left(C_{2}H_{5}\right)}_{2}+H_{2}O_{2}\to \mathrm{OS}{\left(C_{2}H_{5}\right)}_{2}+H_{2}O,$$

$$2C_{3}H_{7}{\mathrm{NH}}_{2}+{\text{COCl}}_{2}\to \mathrm{CO}{\left({\mathrm{NHC}}_{3}H_{7}\right)}_{2}+2\mathrm{HCl},$$

$$\text{trans‐}2\text{‐pentene}+{\mathrm{Cl}}_{2}\to C_{2}H_{5}{\text{CClCHCH}}_{3}+\mathrm{HCl},$$

$$2\text{‐pentyne}+\mathrm{HCl}\to C_{2}H_{5}{\text{CClCHCH}}_{3},$$

$$\text{propylfurane}+H_{2}S\to \text{propylthiophene}+H_{2}O,$$

$${\left(C_{3}H_{7}S\right)}_{2}+H_{2}\to 2C_{3}H_{7}\mathrm{SH},$$

which are reactions 4, 10, 18, 20, 21, 28 and 38 from the FH test set, respectively.^32^ The individual HF and DF‐HF reaction energies are tabulated in Data S1 and summary statistics of the density fitting errors are presented in Tables 6 and 7 for the cc‐pV(*n* + d)Z and cc‐pV*n*Z‐F12 families of orbital basis sets, respectively.

**TABLE 6:** Density fitting errors (kcal mol^−1^) in the HF energy for the seven reactions from the FH test set that contain second‐row elements. The cc‐pV(*n* + d)Z family of orbital basis sets are used and the cc‐pV*n*Z‐JKFit auxiliary sets are used for non‐second‐row elements

Orbital basis	JKFit	MAE	*σ*	MAX
cc‐pV(D + d)Z	cc‐pVDZ	0.14	0.14	0.42
	cc‐pV(D + d)Z	0.11	0.09	0.29
cc‐pV(T + d)Z	cc‐pVTZ	0.07	0.08	0.23
	cc‐pV(T + d)Z	0.02	0.01	0.04
cc‐pV(Q + d)Z	cc‐pVQZ	0.04	0.05	0.14
	cc‐pV(Q + d)Z	0.00	0.00	0.01
cc‐pV(5 + d)Z	cc‐pV5Z	0.03	0.05	0.14
	cc‐pV(5 + d)Z	0.00	0.00	0.00

**TABLE 7:** Density fitting errors (kcal mol^−1^) in the HF energy for the seven reactions from the FH test set that contain second‐row elements. The cc‐pV*n*Z‐F12 family of orbital basis sets are used. The cc‐pV([*n* + 1] + d)Z‐JKFit sets are combined with cc‐pV(*n* + 1)Z‐JKFit for non‐second‐row elements

Orbital basis	JKFit	MAE	*σ*	MAX
cc‐pVDZ‐F12	cc‐pVDZ	0.15	0.12	0.39
	cc‐pV(T + d)Z	0.02	0.01	0.04
cc‐pVTZ‐F12	cc‐pVTZ	0.07	0.09	0.26
	cc‐pV(Q + d)Z	0.00	0.00	0.01
cc‐pVQZ‐F12	cc‐pVQZ	0.04	0.06	0.17
	cc‐pV(5 + d)Z	0.00	0.00	0.00

From Table 6 it can be seen that the density fitting errors in the reaction energies are typically small, even when using the cc‐pV*n*Z‐JKFit auxiliary sets. However, in applications where high‐accuracy is required these errors may be significant. It is also evident that the density fitting errors for cc‐pV(5 + d)Z with cc‐pV5Z‐JKFit are essentially the same as those for cc‐pV(Q + d)Z with cc‐pVQZ‐JKFit, and the MAX density fitting error of 0.14 kcal mol^−1^, in this context, is large and of a similar magnitude to any residual BSIE in the HF reaction energy. We note that when the cc‐pV*n*Z‐JKFit auxiliary sets are used that the MAX error always occurs for the reaction C_4_H_9_SO_2_H + H_2_O_2_ → C_4_H_9_SO_3_H + H_2_O (reaction 4 in the FH numbering scheme), lending additional weight to Ma et al. using this as a test case.^23^ The modifications to the auxiliary sets developed in this work reduce the density fitting errors to very small values for TZ‐5Z, and for QZ and 5Z any remaining density fitting errors in HF reaction energies are typically less than a hundredth of a kcal mol^−1^. At the DZ level the MAX error is 0.29 kcal mol^−1^, but as the conventional HF/cc‐pV(D + d)Z reaction energy is 11.85 kcal mol^−1^ from the analogous cc‐pV(5 + d)Z energy, this is unlikely to be problematic. Addition of the g function in the cc‐pV(D + d)Z + 1 g‐JKFit auxiliary basis reduces the MAX error to 0.15 kcal mol^−1^.

Table 7 shows the density fitting errors in the HF reaction energies when the cc‐pV*n*Z‐F12 orbital basis sets are used with either the cc‐pV*n*Z or cc‐pV([*n* + 1] + d)Z ‐JKFit auxiliary basis sets. For the latter, the cc‐pV(*n* + 1)Z‐JKFit sets are used for non‐second‐row elements. It is important to emphasize here that these results, and those presented in Tables S12–S14 of the SM, are at the HF level and there are no contributions from F12 methodology. However, as mentioned above, the cc‐pV*n*Z‐F12 family of basis sets have more functions than the conventional correlation consistent basis set of the same cardinal number. A comparison of the conventional (not density fitted) HF reaction energies in the SM indicates that, on average, the cc‐pV*n*Z‐F12 orbital basis sets produce energies close to those from the cc‐pV([*n* + 1] + d)Z orbital basis. Based on this, it seems a reasonable expectation that robust density fitting with the cc‐pV*n*Z‐F12 orbital sets should also have similar DF errors as the cc‐pV([*n* + 1] + d)Z orbital sets paired with cc‐pV([*n* + 1] + d)Z‐JKFit. For example, the cc‐pVTZ‐F12 DF error should be similar to the error observed for cc‐pV(Q + d)Z with cc‐pV(Q + d)Z‐JKFit in Table 6. It is clear from Table 7 that combining the cc‐pV*n*Z‐F12 orbital basis with the cc‐pV([*n* + 1] + d)Z‐JKFit auxiliary basis achieves this expectation and hence this becomes our recommendation.

### 3.3 Spectroscopic constants for the diatomic molecule SO

As a final validation of the new JKFit auxiliary basis sets, the density fitting errors in the HF level spectroscopic constants for the diatomic molecule SO are presented in Table 8. SO was chosen here as the prototypical small molecule requiring additional tight‐d functions in the orbital basis.^30^ Initially focusing on the DF errors with the existing cc‐pV*n*Z‐JKFit auxiliary sets, the errors in the computed spectroscopic constants are relatively small, but the errors at the QZ and 5Z level are the same, rather than decreasing for 5Z, as may be expected. An error of almost 0.1 kcal mol^−1^ in *D*_e_ may also be unacceptably large when considering high‐accuracy applications, which is a common usage for 5Z basis sets. The newly developed cc‐pV(*n* + d)Z‐JKFit auxiliary sets reduce the density fitting error at every *ζ*‐level, there is a reduction in error as the size of the basis increases, and the errors are entirely negligible at the 5Z level. As observed in other benchmarks above, the error at the DZ level with the new auxiliary sets is only a minor improvement over cc‐pVDZ‐JKFit, but the BSIE at this level is large; approximately 15 kcal mol^−1^ for *D*_e_.

**TABLE 8:** Density fitting errors in the HF spectroscopic constants of the ^3^∑^−^ state of the diatomic molecule SO

Orbital basis	JKFit	*D*_e_ error	*r*_e_ error	*ω*_e_ error
		(kcal mol^−1^)	(Å)	(cm^−1^)
cc‐pV(D + d)Z	cc‐pVDZ	−0.29	0.0004	−0.8
	cc‐pV(D + d)Z	−0.23	0.0002	−0.7
cc‐pV(T + d)Z	cc‐pVTZ	−0.17	0.0004	−0.7
	cc‐pV(T + d)Z	−0.04	0.0001	−0.1
cc‐pV(Q + d)Z	cc‐pVQZ	−0.09	0.0002	−0.3
	cc‐pV(Q + d)Z	−0.01	0.0000	−0.1
cc‐pV(5 + d)Z	cc‐pV5Z	−0.09	0.0002	−0.3
	cc‐pV(5 + d)Z	0.00	0.0000	0.0

As the correlation consistent basis sets are often used in post‐HF calculations, the impact of the choice of auxiliary basis in the DF‐HF reference on post‐HF energies or properties is also of interest. The density‐fitted Møller–Plesset second order perturbation theory (DF‐MP2)^8^ spectroscopic constants for SO are presented in Table 9, where the DF‐HF reference was calculated either with the existing cc‐pV*n*Z‐JKFit auxiliary sets or the new cc‐pV(*n* + d)Z‐JKFit. The cc‐pV*n*Z/MP2Fit auxiliary basis of Weigend is used in the DF‐MP2 correlation treatment,^44^ with *n* matching the cardinal number of the orbital basis set. This use of DF‐MP2 on a DF‐HF reference introduces a second potential source of error relative to a conventional MP2 calculation, but is also indicative of the errors that may be observed in a typical application. A comparison of Table 9 with Table 8 shows that the respective errors in the spectroscopic constants are similar at a given zeta‐level, albeit with a slight reduction in *D*_e_ error. Using the newly developed cc‐pV(*n* + d)Z‐JKFit auxiliary sets in the DF‐HF reference does reduce the overall error in the DF‐MP2 spectroscopic constants, relative to the analogous value when cc‐pV*n*Z‐JKFit is used. When the newly developed auxiliary sets are used, the error in the spectroscopic constants is below the precision reported in Table 9 at the QZ level, and is effectively negligible at TZ.

**TABLE 9:** Errors in the DF‐MP2 spectroscopic constants of the ^3^∑^−^ state of the diatomic molecule SO due to the choice of JKFit auxiliary basis used in the DF‐HF reference. The cc‐pV*n*Z/MP2Fit auxiliary basis, where *n* matches the cardinal number of the orbital basis set, is used in the DF‐MP2 correlation treatment

Orbital basis	JKFit	*D*_e_ error	*r*_e_ error	*ω*_e_ error
		(kcal mol^−1^)	(Å)	(cm^−1^)
cc‐pV(D + d)Z	cc‐pVDZ	−0.18	0.0003	−1.0
	cc‐pV(D + d)Z	−0.14	0.0000	−0.8
cc‐pV(T + d)Z	cc‐pVTZ	−0.09	0.0004	−0.4
	cc‐pV(T + d)Z	0.00	0.0000	0.2
cc‐pV(Q + d)Z	cc‐pVQZ	−0.05	0.0002	−0.2
	cc‐pV(Q + d)Z	0.00	0.0000	0.0
cc‐pV(5 + d)Z	cc‐pV5Z	−0.06	0.0003	−0.3
	cc‐pV(5 + d)Z	0.00	0.0000	0.0

## 4 CONCLUSIONS

Auxiliary basis sets for use in the density fitting Hartree–Fock approximation have been developed for the second‐row elements Na–Ar, specifically for use with the correlation consistent cc‐pV(*n* + d)Z (*n* = D − 5) orbital basis sets, where the +d indicates the inclusion of an additional set of d‐type functions required for accurate calculations on these elements. This was achieved through the addition of “tight” 1f1g functions to the existing cc‐pV*n*Z‐JKFit auxiliary sets, and re‐optimization of all of the f‐ and g‐type exponents. The exception to this is cc‐pV(D + d)Z‐JKFit, which is created by simply removing all g‐type functions from cc‐pV(T + d)Z‐JKFit. For sodium and magnesium, the cc‐pV*n*Z‐JKFit auxiliary sets were also optimized in this work, and aug‐cc‐pV(*n* + d)Z‐JKFit sets for use with diffuse augmented orbital basis sets were produced for Na and Mg, and revised from existing aug‐cc‐pV*n*Z‐JKFit sets for Al–Ar. Benchmarking of the newly developed sets for molecular energies, reaction energies and the spectroscopic constants of the SO molecule show that density fitting errors are reduced compared to using the cc‐pV*n*Z‐JKFit auxiliary sets, and that the error reduces as the basis set cardinal number increases.

The use of the cc‐pV(*n* + d)Z‐JKFit sets alongside cc‐pV*n*Z‐F12 orbital sets was explored to determine the magnitude of errors that may result from the DF‐HF component of an F12 calculation. To ensure high‐accuracy, we recommend that cc‐pV*n*Z‐F12 orbital sets are paired with cc‐pV([*n* + 1] + d)Z‐JKFit, which results in DF errors roughly equal to those from the cc‐pV([*n* + 1] + d)Z (with matching JKFit) sets that have a similar level of BSIE at the HF level. For F12 local correlation methods on very large systems, some practitioners may desire fewer functions in the auxiliary basis and further data to inform choice of JKFit basis is provided in the SM.

## Supporting information


**Data S1**. Supporting Information.

## Data Availability

The data that supports the findings of this study are available in the supplementary material of this article.
