# Supplementary material for: Correlation consistent auxiliary basis sets in density fitting Hartree–Fock: The atoms sodium through argon revisited
Source: J Comput Chem. 2023 Jan 13;44(11):1119–28. doi: 10.1002/jcc.27069 (PMC10947126; doi:10.1002/jcc.27069)
Supplement: Supplementary file 1 — Data S1. Supporting Information. [file JCC-44-1119-s001.pdf]

Supporting Material: Correlation consistent auxiliary basis sets in density fitting Hartree-Fock. The atoms sodium through argon revisited.

Harry W. Nash, Robert A. Shaw and J. Grant Hill*

*Department of Chemistry, University of Sheffield, Sheffield S3 7HF, U.K. Email: grant.hill@sheffield.ac.uk

1 Composition of diffuse augmented auxiliary sets

Table S1: Composition of the diffuse augmented JKFit basis sets developed in this work [aug-cc-pV(n+d)Z-JKFit] compared to the existing aug-cc-pVnZ-JKFit. For Al–Ar, only the most diffuse f exponent in the new auxiliary basis set differs from the existing sets.

Orbital basis	aug-cc-pVnZ-JKFit	aug-cc-pV(n+d)Z-JKFit
aug-cc-pV(D+d)Z	(14s12p10d4f)	(14s12p10d5f)
aug-cc-pV(T+d)Z	(14s12p10d4f2g)	(14s12p10d5f3g)
aug-cc-pV(Q+d)Z	(14s12p10d5f3g2h)	(14s12p10d6f4g2h)
aug-cc-pV(5+d)Z	(14s12p10d5f4g3h2i)	(14s12p10d6f5g3h2i)

2 Molecular test set

To determine the errors due to density fitting in molecular energies, molecules containing the second-row elements were selected from the test set of Weigend.[1] The BP86/def-SV(P) optimized geometries provided as part of the test set were used in single point calculations. The molecules selected are: Mg_4, $MgCl_2$, MgF, MgH_2, Na_2O, Na_2S, Na_3N, Na_3P, NaCl, NaF NaH, Al_2O_3, Al_2S_3, $AlCl_3$, AlF_3, AlH_3, AlN, Cl_2, ClF, ClF_3, H_2SO_4, H_3PO_4, HCP, HCl, HSH, HSSH, P_2, PF_3, PF_5, PH_3, S_2, S_5, SF_2, SF_4, SF_6, $SiCl_4$, SiF_4, SiH_4, SiO_2 and SiS_2.

3 Molecular energies summary statistics

Tables S2 to S8 present the mean absolute error (MAE), standard deviation (σ) and maximum error (MAX) in the density fitting of the HF energies (ΔDF) over the test set of 41 molecules. In each Table the orbital basis set is fixed.

Table S2: Errors due to the density fitting approximation (mE_h) in Hartree-Fock energies for a test set of 41 second-row element containing molecules using the cc-pV(D+d)Z orbital basis set. The cc-pV(D+d)Z+1g-JKFit set includes an additional g exponent compared to cc-pV(D+d)Z-JKFit, while def2-QZVPP−g-JKFit removes the g functions from def2-QZVPP-JKFit.

Auxiliary basis set	MAE	σ	MAX
cc-pVDZ-JKFit	0.44386	0.48782	1.95343
cc-pV(D+d)Z-JKFit	0.36047	0.41416	1.63820
cc-pV(D+d)Z+1g-JKFit	0.25528	0.31009	1.22989
def2-QZVPP-JKFit	0.11550	0.11362	0.47779
def2-QZVPP−g-JKFit	0.36129	0.37609	1.54011

Table S3: Errors due to the density fitting approximation (mE_h) in Hartree-Fock energies for a test set of 41 second-row element containing molecules using the cc-pV(T+d)Z orbital basis set.

Auxiliary basis set	MAE	σ	MAX
cc-pVTZ-JKFit	0.22923	0.23352	0.93104
cc-pV(T+d)Z-JKFit	0.09221	0.11953	0.52382
def2-QZVPP-JKFit	0.11449	0.11777	0.48110

Table S4: Errors due to the density fitting approximation (mE_h) in Hartree-Fock energies for a test set of 41 second-row element containing molecules using the cc-pV(Q+d)Z orbital basis set.

Auxiliary basis set	MAE	σ	MAX
cc-pVQZ-JKFit	0.13433	0.13615	0.50833
cc-pV(Q+d)Z-JKFit	0.02372	0.02846	0.11931
def2-QZVPP-JKFit	0.14117	0.14076	0.55802

Table S5: Errors due to the density fitting approximation (mE_h) in Hartree-Fock energies for a test set of 41 second-row element containing molecules using the cc-pV(5+d)Z orbital basis set.

Auxiliary basis set	MAE	σ	MAX
cc-pV5Z-JKFit	0.11731	0.12197	0.45694
cc-pV(5+d)Z-JKFit	0.01112	0.01070	0.04365
def2-QZVPP-JKFit	0.15202	0.15269	0.61050

Table S6: Errors due to the density fitting approximation (mE_h) in Hartree-Fock energies for a test set of 41 second-row element containing molecules using the cc-pVDZ-F12 orbital basis set. Also shown is the number of functions in each auxiliary basis set for a single second-row atom (N_ABS).

Auxiliary basis set	MAE	σ	MAX	N_ABS
cc-pVDZ-JKFit	0.43603	0.45398	1.61987	112
cc-pV(D+d)Z-JKFit	0.33330	0.36262	1.29665	119
cc-pV(D+d)Z+1g-JKFit	0.22609	0.27219	1.02497	128
cc-pVTZ-JKFit	0.21306	0.21899	0.88707	121
cc-pV(T+d)Z-JKFit	0.07396	0.09856	0.44141	137
aug-cc-pVDZ-JKFit	0.39536	0.39606	1.50166	128
aug-cc-pV(D+d)Z-JKFit	0.29096	0.30155	1.07423	135
aug-cc-pV(D+d)Z+1g-JKFit	0.18905	0.21350	0.80536	144
aug-cc-pVTZ-JKFit	0.17168	0.16571	0.66975	146
aug-cc-pV(T+d)Z-JKFit	0.03947	0.04228	0.15179	162

Table S7: Errors due to the density fitting approximation (mE_h) in Hartree-Fock energies for a test set of 41 second-row element containing molecules using the cc-pVTZ-F12 orbital basis set. Also shown is the number of functions in each auxiliary basis set for a single second-row atom (N_ABS).

Auxiliary basis set	MAE	σ	MAX	N_ABS
cc-pVTZ-JKFit	0.26219	0.26061	1.04235	121
cc-pV(T+d)Z-JKFit	0.10147	0.12628	0.56115	137
cc-pVQZ-JKFit	0.14453	0.14754	0.54682	148
cc-pV(Q+d)Z-JKFit	0.02164	0.02616	0.11194	164
aug-cc-pVTZ-JKFit	0.21147	0.20082	0.79838	146
aug-cc-pV(T+d)Z-JKFit	0.06013	0.06399	0.24579	162
aug-cc-pVQZ-JKFit	0.13008	0.12945	0.48075	184
aug-cc-pV(Q+d)Z-JKFit	0.01239	0.01158	0.04407	200

Table S8: Errors due to the density fitting approximation (mE_h) in Hartree-Fock energies for a test set of 41 second-row element containing molecules using the cc-pVQZ-F12 orbital basis set. Also shown is the number of functions in each auxiliary basis set for a single second-row atom (N_ABS).

Auxiliary basis set	MAE	σ	MAX	N_ABS
cc-pVQZ-JKFit	0.15432	0.15733	0.58970	148
cc-pV(Q+d)Z-JKFit	0.02670	0.03258	0.14079	164
cc-pV5Z-JKFit	0.12192	0.12733	0.47449	181
cc-pV(5+d)Z-JKFit	0.01096	0.01052	0.04308	197
aug-cc-pVQZ-JKFit	0.13788	0.13678	0.50558	184
aug-cc-pV(Q+d)Z-JKFit	0.01591	0.01573	0.06179	200
aug-cc-pV5Z-JKFit	0.11313	0.11682	0.43809	230
aug-cc-pV(5+d)Z-JKFit	0.00761	0.00666	0.02706	246

4 Distributions of DF errors in molecular energies

Figures S1 to S4 present violin plots of ΔDF for various auxiliary basis sets with the cc-pVnZ orbital basis sets, where n = D, T, Q and 5, respectively.

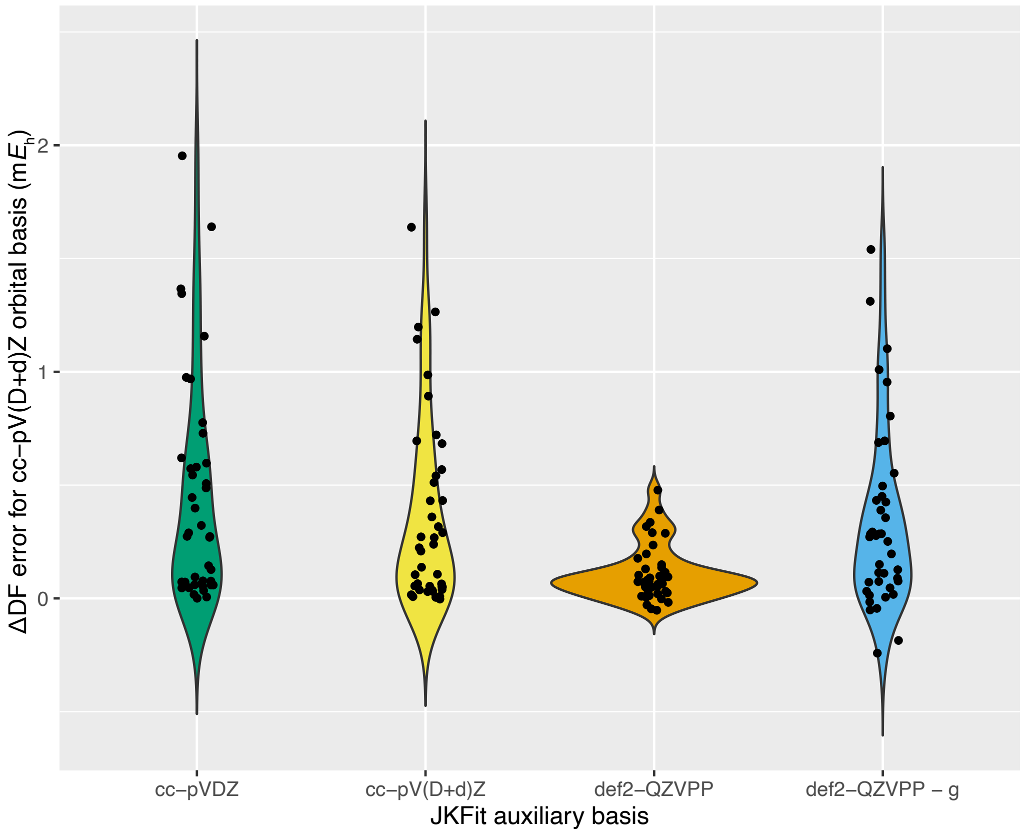

Figure S1: Violin plots of the ΔDF errors (mE_h) in Hartree-Fock energies for a test set of 41 second-row element containing molecules using the cc-pV(D+d)Z orbital basis set. The individual data points with each auxiliary basis set are shown as jitter scatter plots. The def2-QZVPP – g JKFit basis removes the set of g functions from def2-QZVPP-JKFit.

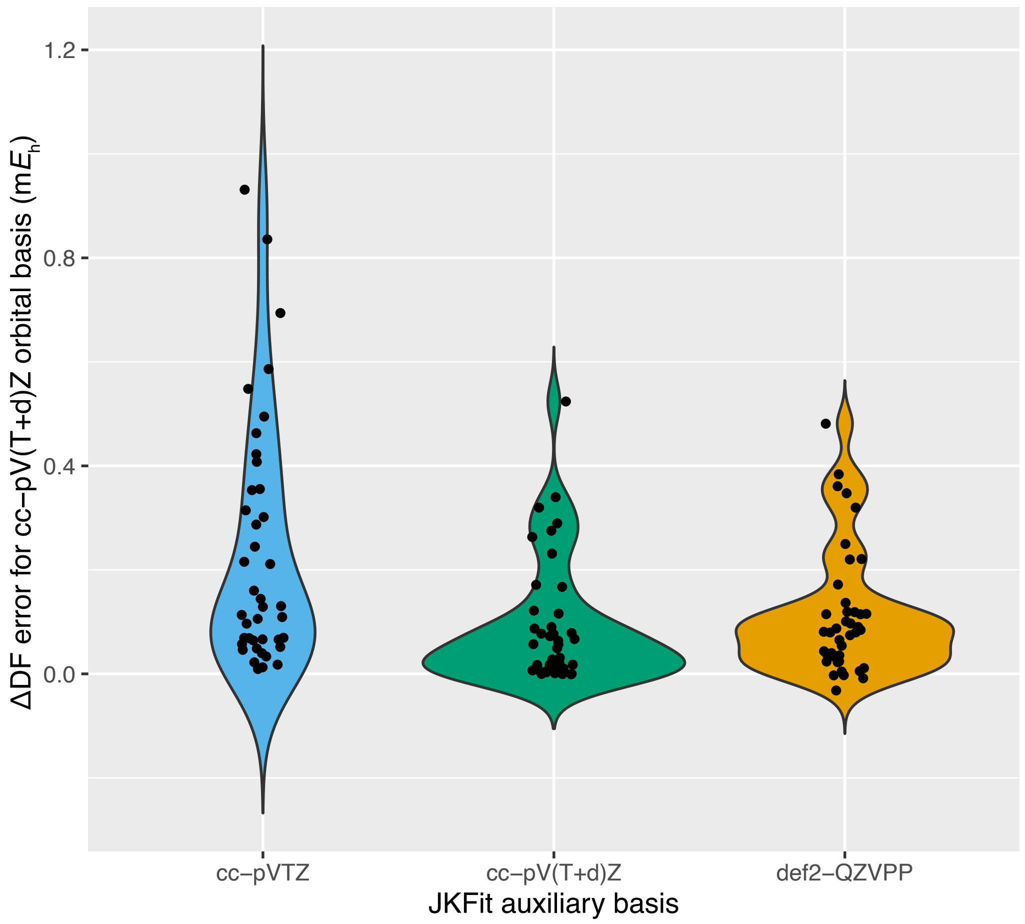

Figure S2: Violin plots of the ΔDF errors (mE_h) in Hartree-Fock energies for a test set of 41 second-row element containing molecules using the cc-pV(T+d)Z orbital basis set. The individual data points with each auxiliary basis set are shown as jitter scatter plots.

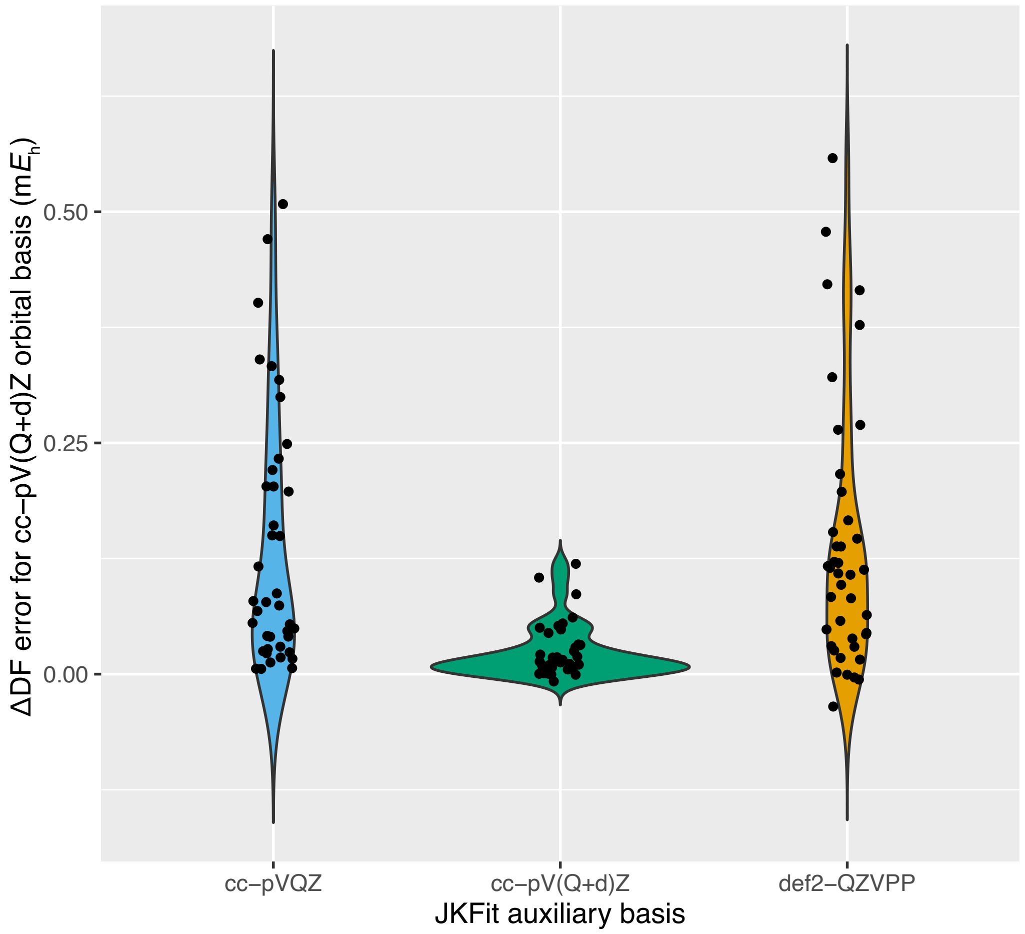

Figure S3: Violin plots of the ΔDF errors (mE_{h}) in Hartree-Fock energies for a test set of 41 second-row element containing molecules using the cc-pV(Q+d)Z orbital basis set. The individual data points with each auxiliary basis set are shown as jitter scatter plots.

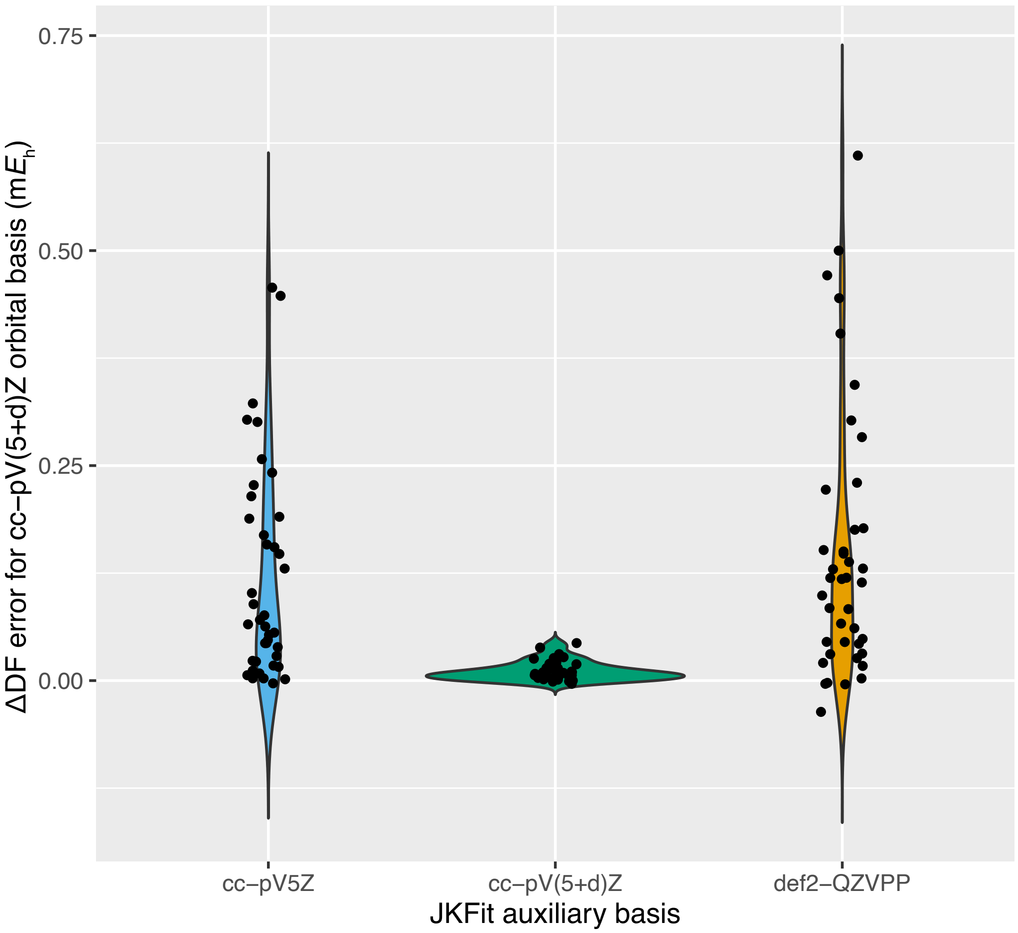

Figure S4: Violin plots of the ΔDF errors (mE_h) in Hartree-Fock energies for a test set of 41 second-row element containing molecules using the cc-pV(5+d)Z orbital basis set. The individual data points with each auxiliary basis set are shown as jitter scatter plots.

Figure S5 shows violin plots distributions of the ΔDF error as the orbital basis set size is increased. The def2-QZVPP-JKFit auxiliary basis set is used in all cases.

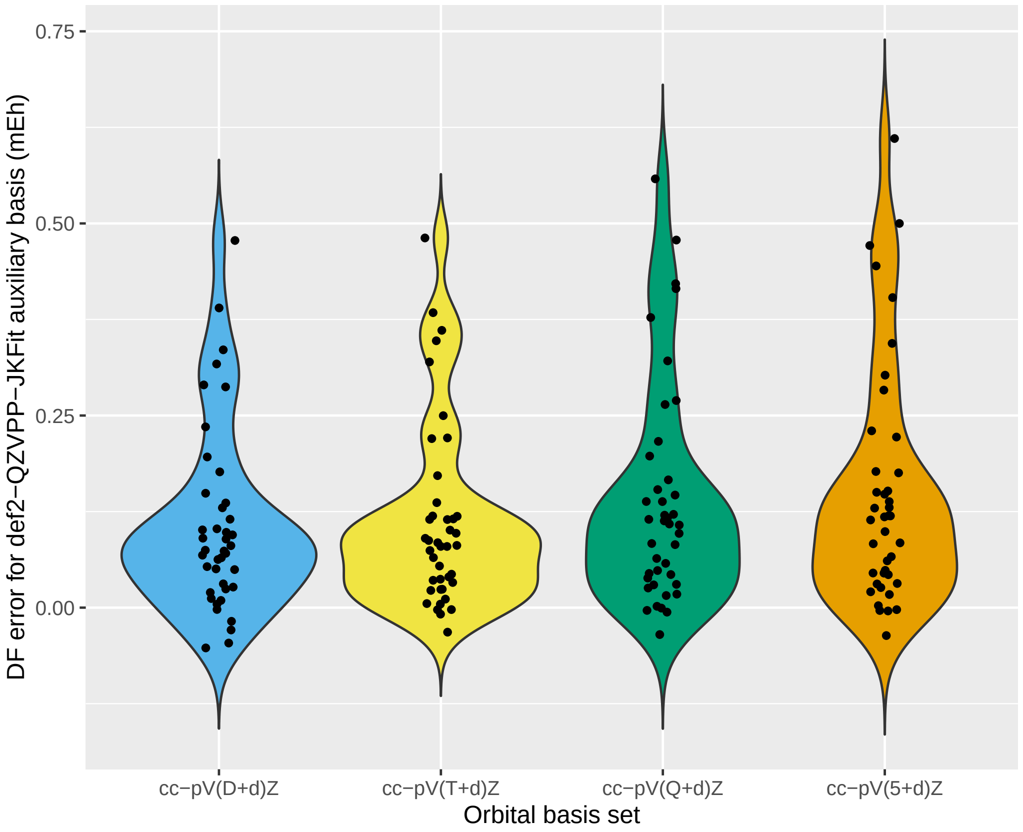

Figure S5: Violin plots of the ΔDF errors (mE_h) in Hartree-Fock energies for a test set of 41 second-row element containing molecules as the orbital basis set is increased. The def2-QZVPP-JKFit auxiliary basis set is used in all cases. The individual data points with each orbital basis set are shown as jitter scatter plots.

Figures S6 and S7 are violin plots showing how distributions of the ΔDF errors change as the orbital basis set size is increased. The cc-pVnZ-JKFit auxiliary basis set family, where the cardinal numbers of the orbital and

auxiliary basis sets match, is used in Figure S6, and cc-pV(n+d)Z-JKFit used in Figure S7.

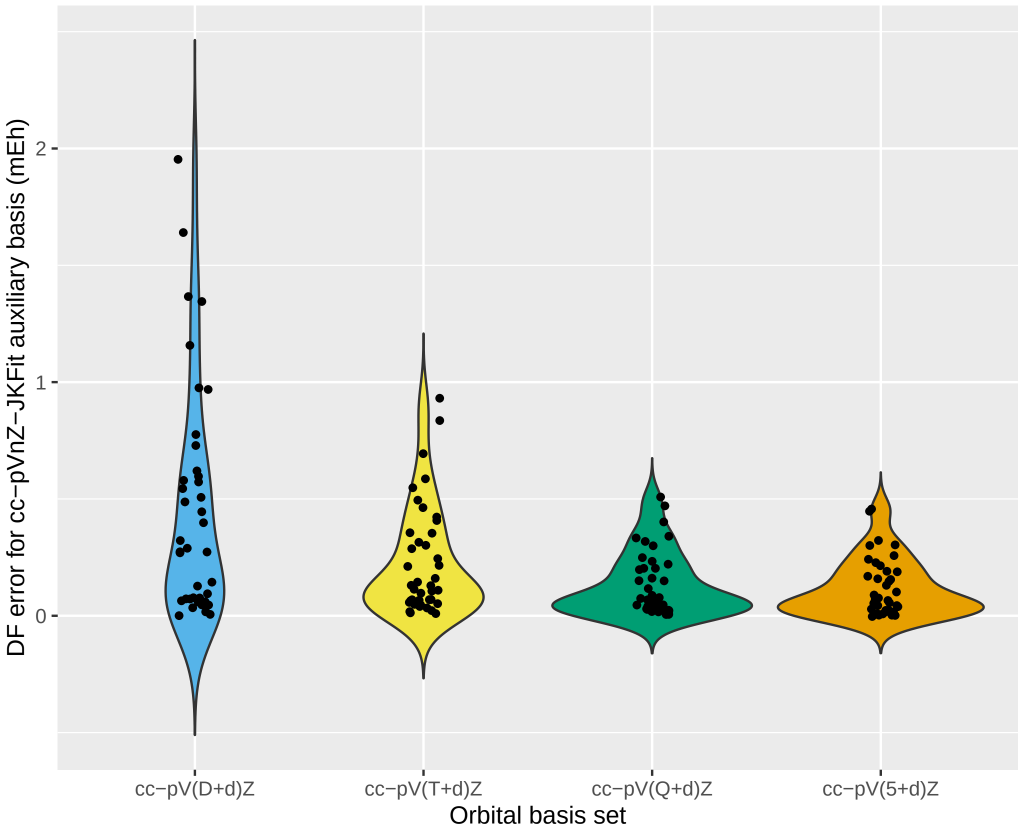

Figure S6: Violin plots of the ΔDF errors (mE_h) in Hartree-Fock energies for a test set of 41 second-row element containing molecules as the orbital basis set is increased. The existing cc-pVnZ-JKFit family of auxiliary basis sets are used, with the cardinal number matching that of the orbital basis set. The individual data points with each orbital basis set are shown as jitter scatter plots.

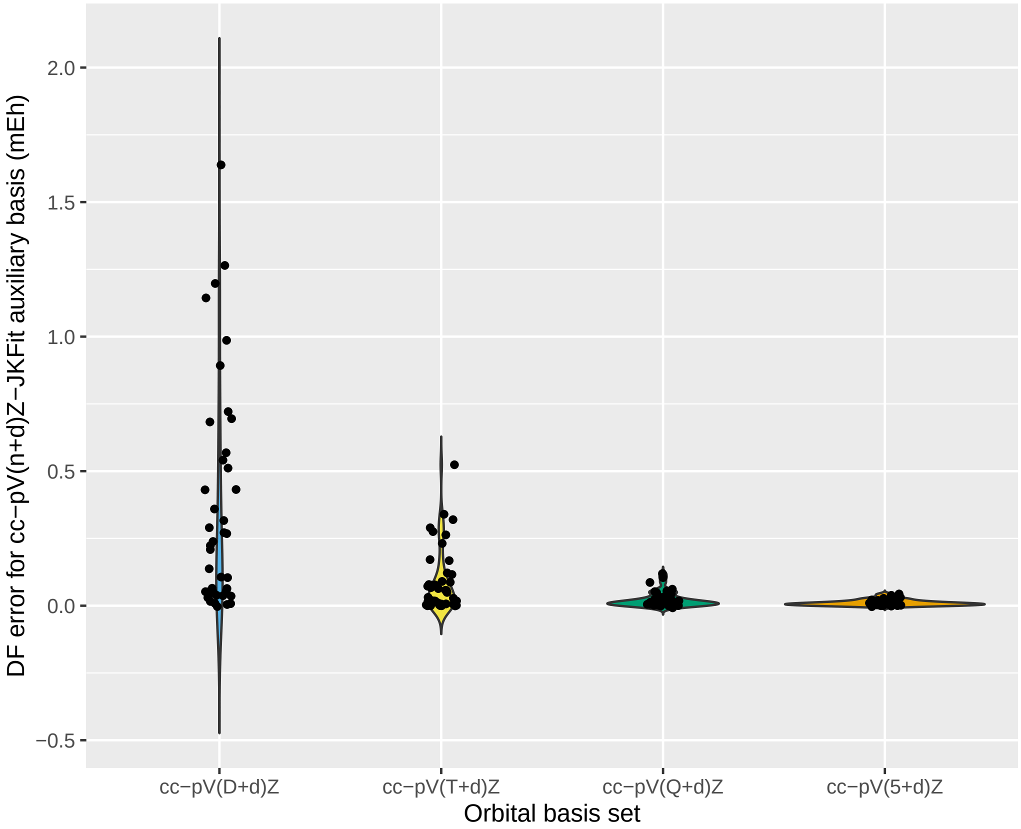

Figure S7: Violin plots of the ΔDF errors (mE_h) in Hartree-Fock energies for a test set of 41 second-row element containing molecules as the orbital basis set is increased. The newly developed cc-pV(n+d)Z-JKFit family of auxiliary basis sets are used, with the cardinal number matching that of the orbital basis set. The individual data points with each orbital basis set are shown as jitter scatter plots.

5 HF reaction energies

Tables S9 to S15 present the HF and DF-HF reaction energies for reactions 4, 10, 18, 20, 21, 28 and 38 from the reaction energies test set of Friedrich and Hänchen.[2] The DF-HF results are presented for two different choices of auxiliary basis. Chemical equations for these reactions are given in the main text.

Table S9: HF and DF-HF reaction energies (kcal mol^{-1}) for the seven reactions from the FH test set that contain second-row elements. The cc-pV(D+d)Z orbital basis set is used.

Reaction number	HF	DF-HF	
		cc-pVDZ-JKFit	cc-pV(D+d)Z-JKFit
4	−75.34	−74.92	−75.06
10	−34.98	−34.76	−34.83
18	−44.33	−44.39	−44.40
20	−32.77	−32.85	−32.86
21	−23.17	−23.11	−23.11
28	−6.94	−6.91	−6.92
38	−18.39	−18.49	−18.48

Table S10: HF and DF-HF reaction energies (kcal mol^{-1}) for the seven reactions from the FH test set that contain second-row elements. The cc-pV(T+d)Z orbital basis set is used.

Reaction number	HF	DF-HF	
		cc-pVTZ-JKFit	cc-pV(T+d)Z-JKFit
4	−85.87	−85.64	−85.84
10	−47.68	−47.56	−47.66
18	−39.57	−39.61	−39.61
20	−28.86	−28.88	−28.89
21	−21.67	−21.66	−21.66
28	−10.55	−10.54	−10.56
38	−15.26	−15.30	−15.29

Table S11: HF and DF-HF reaction energies (kcal mol^{-1}) for the seven reactions from the FH test set that contain second-row elements. The cc-pV(Q+d)Z orbital basis set is used.

Reaction number	HF	DF-HF	
		cc-pVQZ-JKFit	cc-pV(Q+d)Z-JKFit
4	−86.83	−86.69	−86.82
10	−49.69	−49.61	−49.68
18	−38.91	−38.92	−38.92
20	−28.61	−28.61	−28.61
21	−21.40	−21.40	−21.40
28	−11.48	−11.46	−11.48
38	−15.36	−15.37	−15.37

Table S12: HF and DF-HF reaction energies (kcal mol^{-1}) for the seven reactions from the FH test set that contain second-row elements. The cc-pV(5+d)Z orbital basis set is used.

Reaction number	HF	DF-HF	
		cc-pV5Z-JKFit	cc-pV(5+d)Z-JKFit
4	−87.19	−87.05	−87.19
10	−50.41	−50.32	−50.40
18	−38.55	−38.55	−38.55
20	−28.47	−28.47	−28.48
21	−21.35	−21.35	−21.35
28	−11.86	−11.85	−11.86
38	−15.36	−15.36	−15.36

Table S13: HF and DF-HF reaction energies (kcal mol^{-1}) for the seven reactions from the FH test set that contain second-row elements. The cc-pVDZ-F12 orbital basis set is used.

Reaction number	HF	DF-HF	
		cc-pVDZ-JKFit	cc-pV(T+d)Z-JKFit
4	−81.73	−81.34	−81.71
10	−46.38	−46.20	−46.37
18	−38.67	−38.78	−38.71
20	−31.03	−31.15	−31.05
21	−21.35	−21.29	−21.34
28	−11.53	−11.49	−11.53
38	−17.27	−17.39	−17.29

Table S14: HF and DF-HF reaction energies (kcal mol^{-1}) for the seven reactions from the FH test set that contain second-row elements. The cc-pVTZ-F12 orbital basis set is used.

Reaction number	HF	DF-HF	
		cc-pVTZ-JKFit	cc-pV(Q+d)Z-JKFit
4	−86.69	−86.43	−86.68
10	−50.20	−50.06	−50.19
18	−38.85	−38.89	−38.86
20	−28.68	−28.70	−28.69
21	−21.25	−21.24	−21.24
28	−11.70	−11.68	−11.70
38	−15.56	−15.60	−15.56

Table S15: HF and DF-HF reaction energies (kcal mol^{-1}) for the seven reactions from the FH test set that contain second-row elements. The cc-pVQZ-F12 orbital basis set is used.

Reaction number	HF	DF-HF	
		cc-pVQZ-JKFit	cc-pV(5+d)Z-JKFit
4	−87.32	−87.16	−87.32
10	−50.56	−50.46	−50.56
18	−38.55	−38.56	−38.55
20	−28.47	−28.47	−28.47
21	−21.34	−21.34	−21.34
28	−11.93	−11.91	−11.93
38	−15.38	−15.38	−15.38

6 Reference data for the spectroscopic constants of the diatomic molecule SO

Tables S16 and S17 present the conventional (that is, without density-fitting) HF and MP2 spectroscopic constants of the $^3\Sigma^-$ state of the diatomic molecule SO.

Table S16: HF spectroscopic constants of the $^3\Sigma^-$ state of the diatomic molecule SO.

Orbital basis	D_e (kcal mol^{-1})	r_e (Å)	ω_e (cm^{-1})
cc-pV(D+d)Z	39.51	1.4507	1330.8
cc-pV(T+d)Z	53.03	1.4339	1381.4
cc-pV(Q+d)Z	54.79	1.4309	1378.8
cc-pV(5+d)Z	55.39	1.4297	1379.8

Table S17: MP2 spectroscopic constants of the $^3\Sigma^-$ state of the diatomic molecule SO.

Orbital basis	D_e (kcal mol^{-1})	r_e (Å)	ω_e (cm^{-1})
cc-pV(D+d)Z	116.24	1.5404	966.5
cc-pV(T+d)Z	132.65	1.5093	1072.4
cc-pV(Q+d)Z	137.29	1.5032	1087.5
cc-pV(5+d)Z	139.28	1.5005	1093.6

References

1. F. Weigend, J. Comput. Chem. **29**, 167 (2008).

2. J. Friedrich and J. Hänchen, J. Chem. Theory Comput. **9**, 5381 (2013).
